# Is the price right? Paying for value today to get more value tomorrow

**DOI:** 10.1186/s12916-024-03262-w

**Published:** 2024-01-30

**Authors:** Sreeram V. Ramagopalan, Jose Diaz, Grace Mitchell, Louis P. Garrison, Peter Kolchinsky

**Affiliations:** 1 Lane Clark & Peacock LLP, London, UK; 2 Centre for Pharmaceutical Medicine Research, King’s College London, London, UK; 3 Global Health Economics and Outcomes Research, Bristol Myers Squibb, Uxbridge, UK; 4 The Comparative Health Outcomes, Policy, and Economics (CHOICE) Institute, University of Washington, Seattle, USA; 5 RA Capital Management LP, Boston, USA

**Keywords:** Medicine, Drug, Pricing, Cost-effectiveness, Value, Inflation reduction act, Health technology assessment

## Abstract

**Background:**

Contemporary debates about drug pricing feature several widely held misconceptions, including the relationship between incentives and innovation, the proportion of total healthcare spending on pharmaceuticals, and whether the economic evaluation of a medicine can be influenced by things other than clinical efficacy.

**Main body:**

All citizens should have access to timely, equitable, and cost-effective care covered by public funds, private insurance, or a combination of both. Better managing the collective burden of diseases borne by today’s and future generations depends in part on developing better technologies, including better medicines. As in any innovative industry, the expectation of adequate financial returns incentivizes innovators and their investors to develop new medicines. Estimating expected returns requires that they forecast revenues, based on the future price trajectory and volume of use over time. How market participants decide what price to set or accept can be complicated, and some observers and stakeholders want to confirm whether the net prices society pays for novel medicines, whether as a reward for past innovation or an incentive for future innovation, are commensurate with those medicines’ incremental value. But we must also ask “value to whom?”; medicines not only bring immediate clinical benefits to patients treated today, but also can provide a broad spectrum of short- and long-term benefits to patients, their families, and society. Spending across all facets of healthcare has grown over the last 25 years, but both inpatient and outpatient spending has outpaced drug spending growth even as our drug armamentarium is constantly improving with safer and more effective medicines. In large part, this is because, unlike hospitals, drugs typically go generic, thus making room in our budgets for new and better ones, even as they often keep patients out of hospitals, driving further savings.

**Conclusion:**

A thorough evaluation of drug spending and value can help to promote a better allocation of healthcare resources for both the healthy and the sick, both of whom must pay for healthcare. Taking a holistic approach to assessing drug value makes it clear that a branded drug’s value to a patient is often only a small fraction of the drug’s total value to society. Societal value merits consideration when determining whether and how to make a medicine affordable and accessible to patients: a drug that is worth its price to society should not be rendered inaccessible to ill patients by imposing high out-of-pocket costs or restricting coverage based on narrow health technology assessments (HTAs). Furthermore, recognizing the total societal cost of un- or undertreated conditions is crucial to gaining a thorough understanding of what guides the biomedical innovation ecosystem to create value for society. It would be unwise to discourage the development of new solutions without first appreciating the cost of leaving the problems unsolved.

## Background

The pharmaceutical industry’s substantial investment in research and development (R&D) results in a consistent stream of novel medications that not only prolong and preserve life but also alleviate suffering and enhance quality of life. HIV, breast cancer, non-small cell lung cancer, hepatitis C, cystic fibrosis, and multiple sclerosis are examples of diseases where the introduction of novel treatments has made significant improvements in patient length and quality of life [1–8] that is reflected in improved overall population health. Nevertheless, pharmaceutical companies are often accused of exploiting society in their unrelenting pursuit of profits. There is a view among the general public that the price of branded medicines is too high [9]. Critics argue that pharmaceutical companies often set a drug’s price based on what the market will bear rather on what it costs to develop and manufacture it. We believe this belies a misunderstanding of the role of prices in incentivizing investment in an R&D-intensive market. What economists call the “market design” in this situation is based on competition among firms that have *some* monopoly power. However, this “monopoly” is challenged both by time limitations due to the fixed duration of patents and the market entry of competitor products.

Biomedical innovation is driven by the expectation of market-based pricing of branded products for a patent-defined period while made affordable and accessible to the patients who need them through proper insurance, i.e., with low out-of-pocket costs. Markets, patents, and insurance are cultural, not natural, features of our society and economy, rooted in notions of property rights and risk sharing. People conceived of this design to tap into the combination of human ingenuity, drive for financial returns, and aversion to risk to create entire private-sector industries meant to advance the needs and well-being of the community. People can augment this market design when needed (e.g., the government can introduce missing incentives, as with antibiotics, or regulate natural monopolies). They can also dismantle a functioning market when they misunderstand it.

In this article, we aim to provide a viewpoint on several critical aspects of the economics of the pharmaceutical industry. Firstly, we underscore that adequate expected financial returns are necessary to incentivize companies and investors to fund the R&D efforts that are essential to biomedical progress. As pharmaceutical R&D efforts are costly and risky, a promising compound is considered worth investing in when the expected financial return, which we can simplify to the product to two factors—the probability of success and financial value of success—exceeds the cost of development. Of course, prices are just one component of the expected return: sales volume also matters. In addition, investors also need to consider the long-term price trajectory and pricing dynamics following a drug’s launch, which depend on its patent life and competing products. Expected returns are also affected by the cost of making and distributing the drug, as well as public tax policies. When the expected return is too low, either because the price is too low, the volume is too low, the cost of production or distribution is too high, or the duration of adequate pricing is too short, investors stay away, seeking returns elsewhere in the economy.

Secondly, we advocate for a comprehensive evaluation of the value of medicines from a societal perspective as the basis to gauge whether they are worth their cost to society. This evaluation should not be confined to immediate clinical benefits but should be extended to encompass the broad spectrum of advantages conferred by medical interventions in the near and long term to different stakeholders in our society. It is also imperative to incorporate pricing dynamics from competition (both for branded and generic medicines) in this assessment. By adopting this holistic societal approach, the perceived high costs of medicines can be understood and judged in terms of their contributions to the entire economy over the long term, thereby challenging the notion that drug prices are inherently exorbitant. However, for this view to be convincing, we must consider how and why several of the arguments advanced to argue that drugs are overpriced are flawed. For example, arguing that a drug is overpriced if it generates more in profit than it costs to develop ignores the need for a return on investment for an entire portfolio of projects from which few successes emerge. Arguing that drugs are overpriced because patients cannot afford them ignores the important role of insurance plan design in determining drug affordability and access. Also, arguing that drugs are overpriced in the USA because they are cheaper elsewhere fails to consider that other countries are possibly paying too little to support global R&D and are essentially freeriding off the USA’s greater ability and willingness to support biomedical innovation [10].

While it remains appropriate to condemn specific instances of excessive economic “rent-seeking” or even price gouging when companies exploit regulatory or payment system loopholes to keep competitors away, it is wrong to imply that this happens with all medicines. Market flaws are not the same as the whole market being broken: flaws can be fixed without dismantling the whole market.

It is also both naive and harmful to population health to simply point to calculations by HTA bodies and health economists that claim to demonstrate that specific drugs are not cost-effective, without first scrutinizing the underlying methodology to see if it truly reflects all of society’s values. We ask readers to consider how some first principles of value assessments might lead to a broader support for medical innovation.

**Fig. 1:**
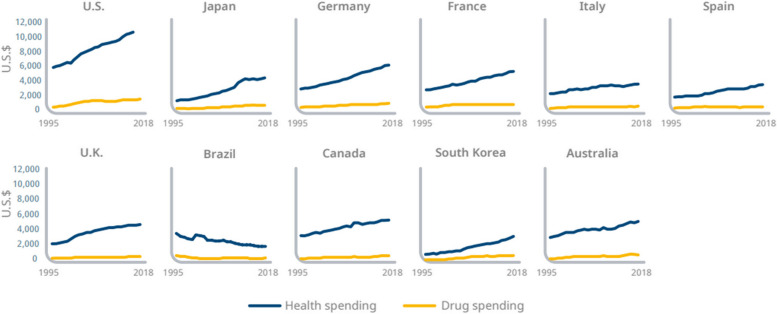
Per capita drug and health spending in real PPP 2020$, 1995–2018. Source: IQVIA Institute for Human Data Science [11]

Lastly, while the discourse surrounding drug pricing is indeed robust and often contentious, it is crucial to note that the lion’s share of healthcare expenditure is not allocated to medicines. Instead, hospital spending constitutes the majority of these costs [11]. Notably, over the last 25 years, overall healthcare spending per capita in the USA based on a real purchasing power parity (PPP) has risen at a consistently higher rate than drug spending, which has remained comparatively stable (Fig. 1). This phenomenon is explained at least in part by the fact that, unlike hospitals, physicians, and everything related to healthcare services, drugs go generic. This is not a reason to not scrutinize the value of medicines but rather to take a broader societal context for assessing their value since one of the major benefits of medicines is that they can keep patients out of hospitals, generating savings where healthcare costs are greatest. Therefore, we have to examine the full range of medical expenditures and arguably even other areas of the economy (e.g., medicines can preserve productivity) to better support the optimal allocation of resources where they can create the most value for society.

## Drug pricing fosters and directs innovation

**Fig. 2:**
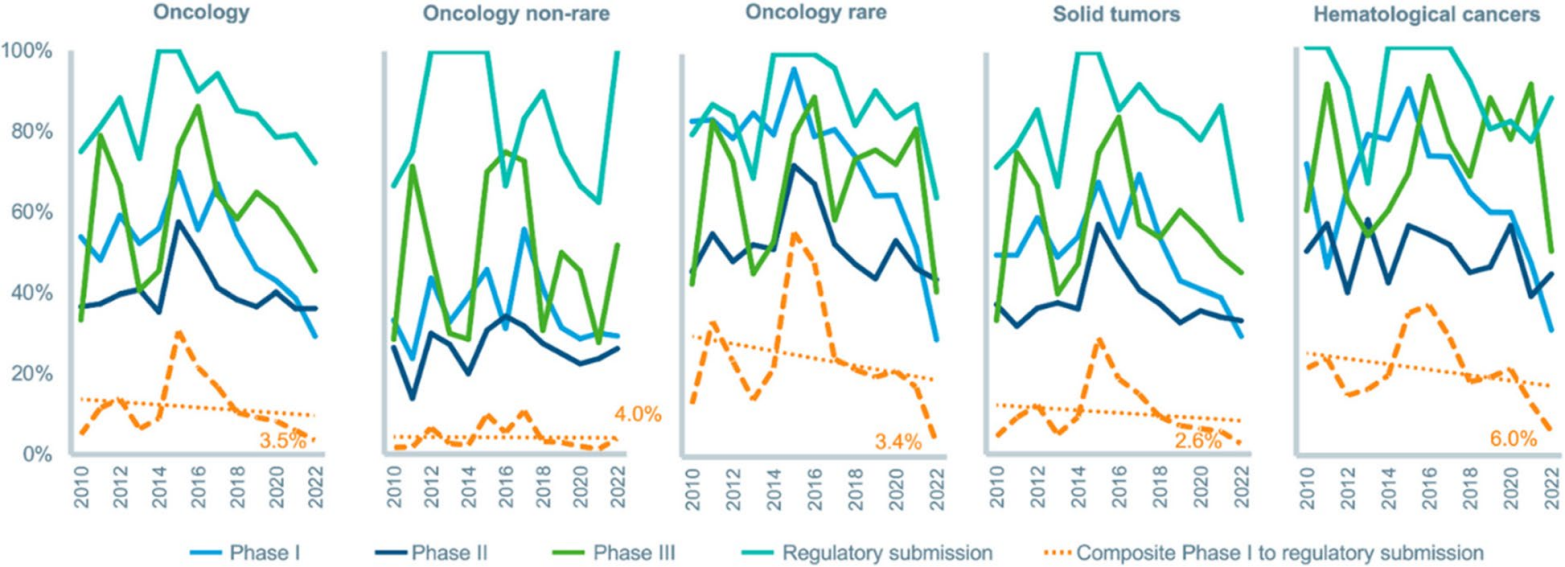
R&D success rates in oncology by therapy area, 2010–2022. Source: IQVIA Institute for Human Data Science [17]

The process of developing a new drug, from preclinical research to market, is time-consuming, risky, and costly, often taking more than a decade and costing billions of dollars [12–16]. The risk is best reflected in the very low probability of a potential drug making it through all stages of clinical trials and securing regulatory approval. Estimates suggest this probability is, on average, less than 10%; however, recent data for oncology show a lower estimate at about 3.5% (Fig. 2) [14]. The high “failure rate” depends on the starting point: for example, probably < 0.1% of all molecules tested in preclinical studies end up being FDA-approved. For those that embark on this journey, the odds of reaching the finish are long, and there is no guarantee that costs incurred for failed products will be recovered in any form. Even if a product is successful in clinical trials and secures regulatory approval, it may not generate enough revenue to recoup the multiple investment attempts that went into its development. Thus, pharmaceutical R&D only starts to make sense at the portfolio level, where the cost of planting many seeds and nurturing a whole garden of projects can only be justified by robust financial returns from selling the few prized fruits.

Understanding the elasticity of innovation—the number of new molecular entities developed in response to market/revenue size—is important. Acemoglu and Linn used population demographic changes to demonstrate that innovation in drug development is partially driven by the size of the potential market (in their analysis of the US population, more drugs were developed for diseases of middle age as this market size increased over time) [18]. Acemoglu and Linn estimated that a 1% increase in the potential market size for a drug leads to a growth of approximately 4% in the entry of new non-generic drugs and new molecular entities [18]. Golec and Vernon analyzed the tangible impacts of price controls, comparing drug pricing and biopharmaceutical company R&D spend in the EU and USA between 1986 and 2004 [19]. They observed that regulations (including HTA) resulted in lower drug prices in the EU and that biopharmaceutical companies which had sales more closely linked to the EU, and therefore received less revenue, spent less on R&D. If the USA had the same pricing as the EU, it was estimated that the consequent lower R&D spend by firms focused on the US market (which includes companies based in the USA and other countries) would have led to 117 fewer new medicines being developed [19].

Interpreting these historical experiments is challenging and while there is no clear consensus among economists on the elasticity of innovation [20], the idea that larger markets (and presumably therefore larger rewards) justify investors taking greater risks is axiomatic in economics, and there is consensus that potential market size (and therefore expected revenue and ultimately profit) influences pharmaceutical innovation [21]. In all industries, financiers will be driven to invest in areas where the expected rate of return is greatest [22]. Policies that reduce rewards for the creation of a novel medicine will inevitably reduce biopharmaceutical investment. So, the issue is whether getting fewer new medicines is an acceptable consequence of such policies or a price we would rather not pay. Although the size of the effect is open to debate, the importance of incentives is clear [21].

It is also clear that market forces direct R&D decisions. There are numerous biopharmaceutical products that the market does not reward, such as novel antibiotics, so there is little investment in developing such products. But where price regulations would cap the rewards that the market is allowed to bestow upon a product that it deems to have a high value, such as an effective drug for brain cancer, the only direction for investment to go is down [23–25].

A key feature of the market design for innovative medicines is the importance of the patent system. While profits earned during the initial patented period are essential to stimulate innovation, these rewards do not—and should not and need not—last forever. Products that are protected by patents and have received regulatory approval have a period of patent and exclusivity protection that averages approximately 14 years [26], during which the manufacturer retains the right to market its compound without competition from generic or biosimilar copies [24]. This is, however, rarely a monopoly on the treatment of a particular disease. Competitors develop their own products in the same drug class and/or for the same indication. As long as a patient has more than one option for standard-of-care treatment, those medicines (assuming they are sold by different companies) can compete on price for market share. An example of this comes from hepatitis C. In late 2013, the FDA approved sofosbuvir (Sovaldi®), a highly efficacious treatment [27, 28]. Despite the substantial increase in the HCV cure rate, markedly improving patient length and quality of life and providing significant health system cost savings, there was significant media attention paid to the drug’s initial list price of $84,000 per course of treatment in the USA [27]. However, as competitor products subsequently launched, price competition for these direct-acting antiviral regimens for hepatitis C treatment markedly drove down the net treatment cost of this drug class [29] to currently about $20,000 per course. This reflects the fact that in the USA, pharmaceutical companies receive the net price for a medicine, which is the list price minus rebates, discounts, and fees. Pharmacy benefit managers (PBMs), who are third-party intermediaries between pharmaceutical manufacturers and insurance providers, keep and profit from part of the discounts or rebates negotiated from pharmaceutical companies.

Ultimately, for most medicines, the profitability of the period of effective market exclusivity is not only tempered by competition with other drugs but will ultimately conclude, after patent expiry, with the launch of generics, or, in the context of biologics, biosimilar versions to compete with the original and other drugs within the class. Starting in 2026, the US Inflation Reduction Act (“IRA”) gave Medicare the ability to force down the price of a medicine 9 years or 13 years after its launch, depending on whether it was approved under the new drug application (“NDA,” e.g., small molecules, small peptides, oligonucleotides) or biologics license application pathway (“BLA,” e.g., enzymes, antibodies, vaccines), respectively [30]. For drugs that would otherwise succumb to price competition from generics or biosimilars after patent expiry, such price regulation does not solve any clear market failure. But some drugs may in effect have a “natural” monopoly: for example, sometimes (a) they are incredibly difficult to manufacture to specifications that the FDA would consider bioequivalent or biosimilar (e.g., cell and gene therapies or drug-device combinations) or (b) trials of generics/biosimilars would be unethical (e.g., in cases where there is a short window to give an effective therapy, such as infantile spasm or when patients with a progressive disease cannot be asked to risk randomization to an unproven agent). A drug may also turn out to have a “natural” monopoly if its market proves to be too small to support more than one manufacturer. If such drugs remain expensive after patent expiry, pricing regulation can aim to achieve the intent of the patent system (e.g., the IRA). Consequently, financial rewards for new medicines are typically limited by on-patent market competition, off-patent generic/biosimilar competition, and price regulation.

## Pricing as a reflection of value

Pricing based on the R&D costs of one approved molecule does not account for all the others that did not make it to the market. Additionally, while we tend to focus on the successful products in the existing R&D ecosystem, the literature has shown that both successful and failed R&D efforts have generated important scientific knowledge that has improved the efficiency of our R&D system [31]. Put simply, it is impossible to innovate without failure, and it is important to recognize that failures are valuable, too, because what we learn—as an R&D ecosystem—from our failures helps us to eventually succeed.

If we agree that providing sufficient rewards is essential to incentivize biopharmaceutical R&D, what should society pay? Firstly, this may seem a strange question to those who operate in the actual market-based system, because the answer they often encounter is that payers try to pay as little as possible. If faced with enough choices, payers, specifically PBMs in the USA, can simply play companies off against one another and take the best offers. Understandably pre-occupied with their own priorities, none of these individual payers is directly concerned with rewarding innovation from the societal perspective. Innovators are constantly trying to invent something uniquely useful in the hope that they will not face too much competition and will therefore have pricing power. In other words, they want to be able to control the supply of a product that buyers want badly enough to pay the price that the innovator sets, and even where there are a few competitors, companies tend to be savvy enough not to trigger a ruinous price war, which is why it often takes loss of exclusivity and many generic competitors entering the market to drive a drug’s price down steeply.

So, the key question should be properly restated to: how high a price should society be willing to pay for a medicine (or any product) when innovators have the power to set a price?

Formalized HTA processes have been employed for many years in a number of countries to determine the value of novel health technologies and, in many cases, to inform reimbursement and pricing decisions. Major HTA organizations include the National Institute for Health and Care Excellence (NICE) in England and Wales, Haute Autorité de Santé (HAS) in France, Gemeinsamer Bundesausschuss (G-BA) in Germany, the Pharmaceutical Benefits Advisory Committee (PBAC) in Australia, and the Canadian Agency for Drugs and Technologies in Health (CADTH) in Canada. These agencies perform HTA on behalf of ministries of health (or equivalent) and therefore tend to take a narrow perspective of healthcare budgets when they perform assessments. Economic evaluation, including cost-effectiveness analysis (CEA), is commonly conducted as part of HTA to analyze the costs and effects of alternative interventions that may be given to a defined population. In traditional CEA, the health benefits of health technologies are typically captured by quality-adjusted life year (QALY)—an outcome measure that integrates both the duration and quality of life [32]. QALYs provide a consistent benefit measure across different treatment options and allow decision-makers to evaluate the cost-effectiveness of novel medicines in different therapeutic areas. A key output from CEA is the incremental cost-effectiveness ratio (ICER): the difference in costs between two competing interventions (usually the innovative technology being evaluated and the standard of care without that novel product) divided by the difference in outcomes—represented as cost per QALY [26]. A number of HTA bodies have an implicit or explicit ICER threshold, whereby medicines that have an ICER below this are considered cost-effective and more likely to be reimbursed. When deemed not cost-effective based on HTA, medicines may be blocked by a country or else their entry to a market may be delayed; consider that while 85% of new medicines launched anywhere in the world between 2012 and 2021 were available in the USA, that figure was 61% in Germany, 59% in the UK, and 52% in France [33], and delays in access to medicines among European countries can be substantial, for example, averaging from an average of 100 days in Germany to 800 days in Poland [34].

Conventional CEA has, however, important known limitations and often reflects only a portion of the benefits that stem from a healthcare intervention [35]. New medicines not only benefit patients, but also their family members and society at large. For example, the impacts of improved health on a patient’s ability to return to work are not captured in their QALY gains but have benefits for a patient and society (e.g., taxes paid by the patient and disability allowances not paid). While the evidence base is not complete, one study looking at a time period from 2000 to 2015 has quantified that medicines increased wages by $233 billion annually in the USA [36], which is roughly in line with what the USA spent per year on average on all branded pharmaceuticals during that time [37]. Additionally, some medical innovations provide benefits over current treatment options, such as more convenient dosing regimens or alternative administration methods (for example, subcutaneous at-home treatment versus intravenous at-hospital treatment). These benefits may accrue to the patient (i.e., need to take medicines less frequently, reduced medical travel costs) and also spill over to society (increased adherence to treatment of schizophrenia reduces risk of psychotic episodes that might otherwise end with the patient being jailed, for example) [38, 39].

**Fig. 3:**
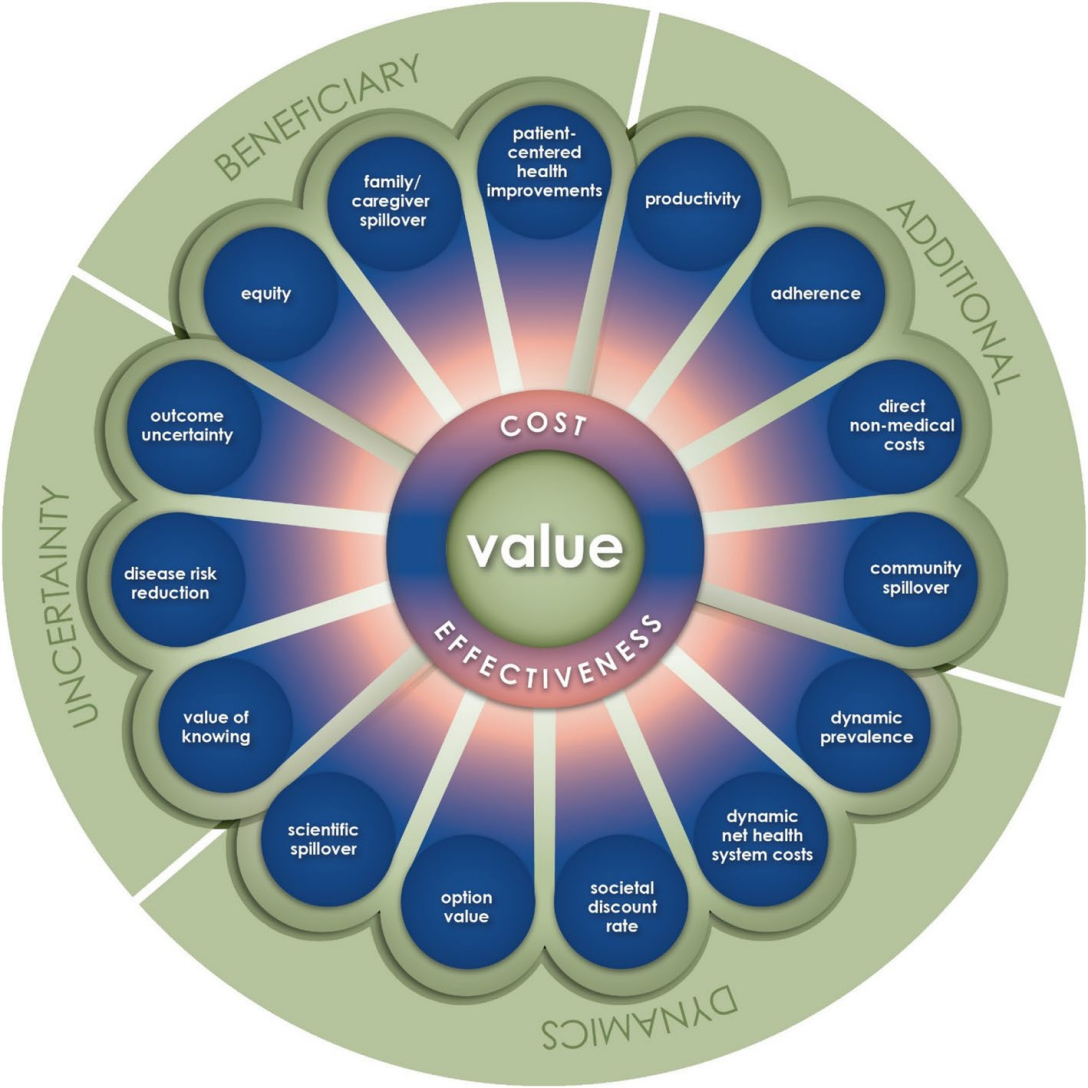
Source: Modified ISPOR Value Flower (modified from Neumann et al. [44], graphic borrowed with permission from No Patient Left Behind)

**Fig. 4:**
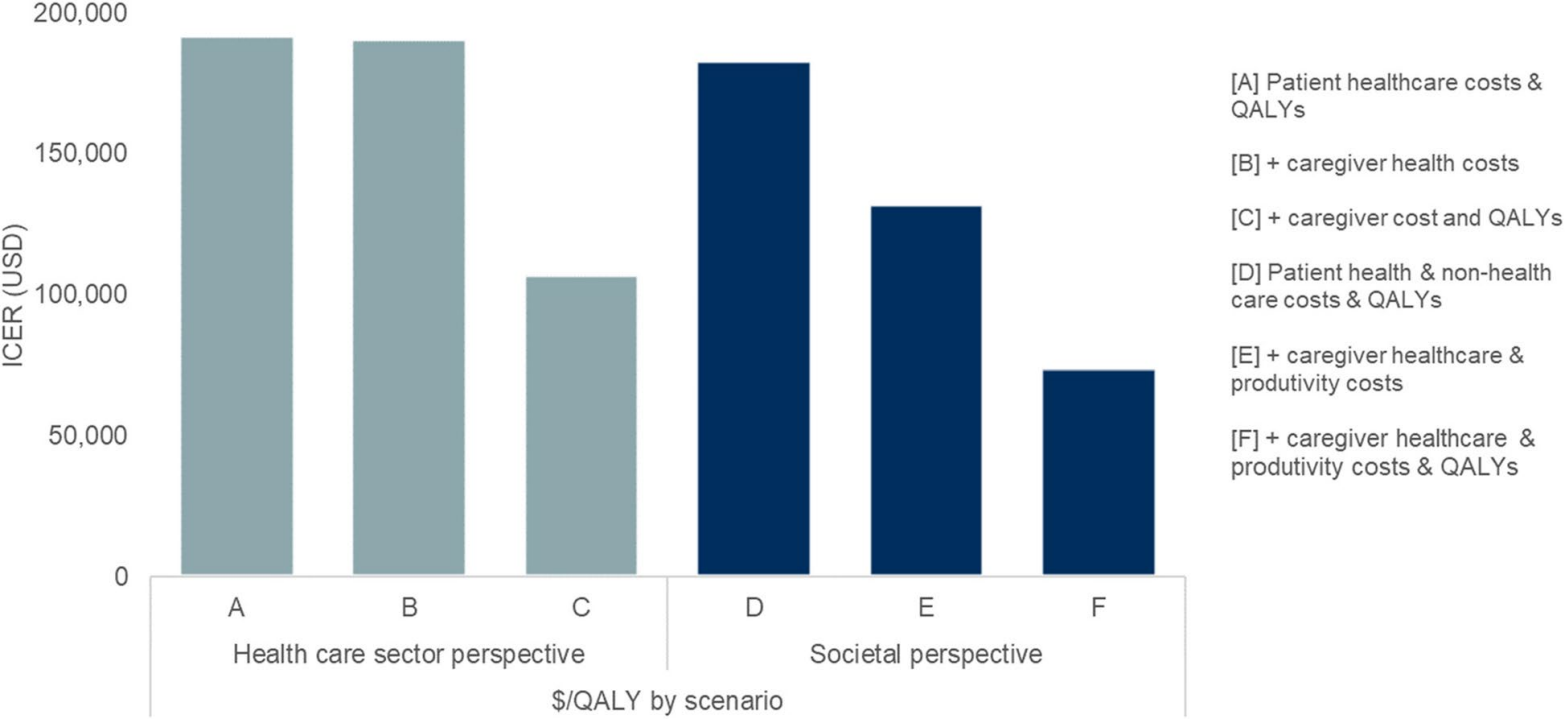
The impact of including broader elements of value on the incremental cost-effectiveness ratio. Source: Modified from Ito et al. [45]. Ito and colleagues estimated the cost-effectiveness of a hypothetical treatment for Alzheimer’s disease that delays progression to dementia for patients with mild cognitive impairment [45]. The ICER varied depending on whether the model included factors beyond a patient’s own health. When caregiver costs (e.g., caregiver time caring for the patient or work lost by taking care of the patient) and caregiver quality-of-life effects were included, the cost-effectiveness of the hypothetical Alzheimer’s treatment improved substantially, from $183,000 per QALY gained to $74,000 per QALY gained—a 60% decrease in the cost-effectiveness ratio. At a decision threshold of US $150,000 per QALY gained, this study demonstrated that failing to consider important value elements from a societal perspective can underestimate the value of novel medicines, impact coverage and reimbursement, limit access, and reduce the welfare of patients [46]

The ISPOR Value Flower, originating from the work of the ISPOR Special Task Force in 2018, was a value framework that delineated several of these broader and non-traditional elements, reflecting a fuller range of benefits associated with healthcare interventions, including “petals” such as productivity gains, scientific spillovers, financial and health risk protection, value of hope, and option value [40] (Fig. 3). Subsequently, numerous studies have shown that, by using this framework to incorporate these additional elements of value into CEA, the resulting ICER can change dramatically (an example seen in Fig. 4). The incorporation of broader value elements into CEA can be technically challenging and can introduce additional uncertainty, as we lack established methods and quality data sources to measure some of them, such as scientific spillovers [40]. This is perhaps why CEA that considers several of these additional value elements are rarely performed or have only considered a very limited set of these elements to date. This is also compounded by the fact that many HTA agency guidelines do not provide explicit recommendations for incorporating broader value elements [41]. This may be because the remit of a HTA agency is often more narrowly focused on the healthcare sector, and value elements from a broader societal perspective might not be directly accrued to those stakeholders in the healthcare sector [42]. In other words, they may care more about the budget of the payer they are advising than value to society as a whole. However, not evaluating all potential elements of value leads to an inaccurate and potentially underestimated valuation of health interventions from a societal perspective [43], which has direct consequences for patients when they are denied access based on HTA. Furthermore, by underutilizing what have actually been cost-effective medicines, society reduces incentives for the development of more such medicines that would have likewise been worth their risk-adjusted costs of development and the prices—though really the profits—to incentivize those investments. Improving the math by which we value drugs could allow us to appreciate that the market has actually been getting value for society and can be relied upon to effectively elicit bargain biomedical breakthroughs.

## Cost effectiveness analyses need to factor life cycle drug pricing

**Fig. 5:**
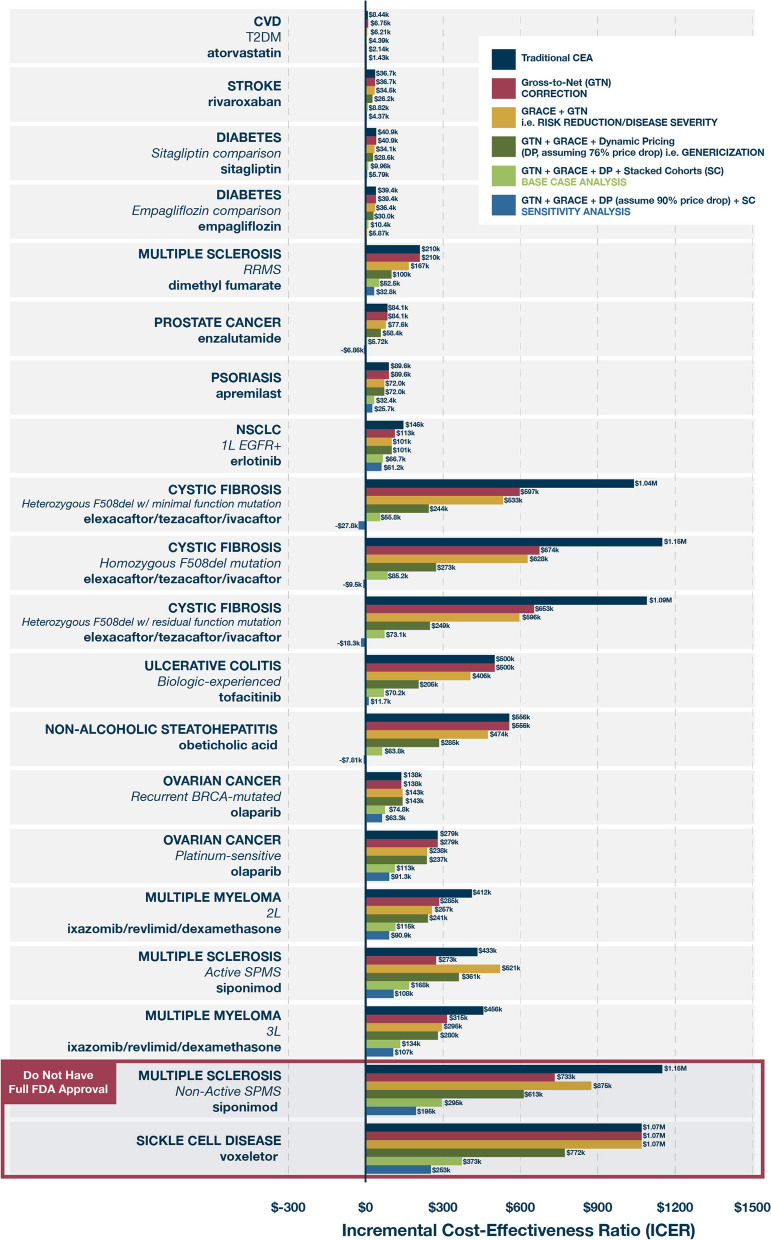
The effect of life cycle pricing on cost-effectiveness analyses. Source: Lakdawalla et al. [51] (graphic borrowed with permission from No Patient Left Behind). Lakdawalla et al. reviewed a set of 20 traditional CEAs published by the US-based Institute for Clinical and Economic Review and accounted for several additional values as well as dynamic pricing [51]. Importantly, they used a “stacked cohort” model to account for patients starting treatment in years post-launch, including patients who start treatment after a drug has gone generic and therefore received extremely inexpensive benefits. Their base case assumption was that drugs would drop in price by 76%, based on the average decline of a historical set of medicines after their loss of exclusivity. However, recognizing that many of the drugs ICER was evaluating were outliers more likely to drop by more than that because they are specialty drugs for orphan disorders (e.g., the cancer drug Tarceva and the multiple sclerosis treatment Tecfidera dropped in price by over 98% after going generic), they included a sensitivity analysis that assumed a 90% price drop after 14 years [51]. Of the 20 drugs, traditional CEA had deemed only 8 to be cost-effective. But the more inclusive generalized CEA approach showed that 17 were cost-effective in the base case, and 18 were cost-effective in the sensitivity analysis. Interestingly, two remained above the cost-effectiveness threshold even in the sensitivity analysis. One was a drug that had failed its clinical trial and was not even approved for the indication that ICER had modeled (in other words, no one is asking society to pay for it). The other was a drug for sickle cell disease that was approved on an accelerated basis based on a biomarker and is still undergoing outcomes studies that would inform its cost-effectiveness. Lakdawalla et al. pointed out that their calculations only accounted for some of the traditionally missing values of these medicines and that adding in other elements, such as caregiver spillover, would likely further improve their cost-effectiveness

In addition to ignoring the values already discussed, traditional CEA often ignores how a product’s price changes during its time on the market, assuming a constant price (usually set to be the launch price) over the patient’s lifetime or modeling horizon. This approach in effect overestimates the cost of treatment as it does not account for the price reductions resulting from on-brand competition or following generic entry at patent expiry. Despite the ISPOR Drug Cost Task Force in 2010 stating that “if a 25-year model is prepared, it should reflect the expected market realities of price changes, if any, during the patent-protected period and the impact of generic entry and its related price erosion,” the majority of HTA guidelines for economic evaluation do not address the issue of drug genericization, and only a handful suggest that base-case analyses should incorporate assumptions regarding future alterations in drug prices [47, 48]. The costs of leaving disease treatments unchanged can be forecast more than 25 years into the future, and so therefore should the value of having a medicine that averts those costs, especially when the medicine itself will go generic on average 14 years into those projections. It is noteworthy that the vast majority of published pharmaceutical cost-effectiveness analyses do not make any assumptions about future reductions in drug prices following the loss of a drug’s exclusivity [48]. This omission distorts results by misrepresenting total drug costs and not reflecting real-world conditions (Fig. 5). Trying to capture the value of future alterations in drug prices is undoubtedly a complex area for CEA [49], but it is not that difficult to project—recognizing that all CEA models at launch are projections—given the substantial body of evidence that prices fall dramatically once medicines go off-patent. There is a need for more research and explicit guidance regarding suitable methodologies for contemplating genericization and other fluctuations in drug prices, up or down. However, incorporating genericization will allow society to better use CEA when evaluating a drug’s costs and benefits over timeframes that matter to governments and individuals, even if they fall outside of the short budgetary windows of private-sector payers [50].

In summary, traditional CEA often considers the narrow view of just the patient and healthcare system in the present, ignoring other factors that affect patients, their caregivers, the healthcare system, and society as a whole. Once these factors are incorporated, it may turn out the market forces are finding a price at which many medicines provide considerable value for society’s money. Whether these prices are high enough to keep driving innovation is a separate but related question; they appear to be in most cases judging from current pace of R&D in many therapeutic areas. In a few cases, such as for hospital-administered antibiotics, expected market prices cannot adequately make up for the low volume of prescriptions and therefore expected revenues and profits are too low to spur investment in needed R&D. In these cases, government interventions (e.g., via novel payment models) are needed to address the market failure [52]. But where cost-effective market prices are adequate to incentivize investment in further R&D of cost-effective medicines that will eventually go generic, price controls risk creating a market failure where there is not one.

In our view, pricing regulations should focus on upholding the purpose of the patent system, which is to make sure that drugs become generic or biosimilar after an adequate period of market-based “reward” [53]. The markets may offer a small reward or a large one depending on whether the product is valued by patients, doctors, payors, and society as a whole, but regulations would best not interfere with market-driven pricing during the period intended by the patent system. If medicines have been worth their prices, then it means that the market-based pricing mechanisms that bring about those prices (i.e., many drug companies negotiating with many payers, public and private) have been getting value for society, and it is worth considering the merits of continuing to rely on those mechanisms (rather than overriding them with price controls, as some academics and policymakers propose from time to time) [54] as long as they are generating value for society.

Some would say that it is worth defunding biomedical innovation if the money will be spent in ways that generate even more value, but they then fail to subject all the other uses of that capital to the same cost-effectiveness scrutiny. Consider Alzheimer’s disease: we can either pay temporarily high prices for medicines (that will eventually go generic) in the hopes of incentivizing progress in dementia prevention, or we must accept that we will have to spend more on nursing homes, dementia villages, and considerable caregiver burden, on top of the suffering and indignity of dementia itself. As long as we value life and well-being, the question is how to get the most value for what we are prepared to spend, and the tens of trillions we will spend on healthcare services to manage Alzheimer’s in the coming decades suggest that we might get a much better return on investment if we incentivized R&D to avert it [55].

We recognize the US healthcare system, which provides the majority of funds for pharmaceutical R&D while allocating the largest share of GDP to healthcare of any country, is a complex system with a wide range of broad-based and targeted subsidies for particular activities. Some would argue that spending on new medicines or healthcare spending as a whole is too high versus other social needs [56]. Addressing this question is beyond the scope of this paper. However, we would emphasize spending to support innovative medicines is unique in that (a) it is a small share of total spending, (b) there are clear health gains attributable to new medicines [57], (c) new medicines can go generic and often avert healthcare costs that do not go generic, and (d) the entire world population of 8 billion humans benefits from the availability of curative medicines (e.g., for HIV or HCV) or prophylactic vaccines (e.g., for smallpox and measles). So, even if one were to decide to reduce US spending on healthcare, cutting down spending on innovative medicines does not seem, to us, to be a logical place to start.

## A broader assessment of healthcare spend puts branded drugs in proper context

**Fig. 6:**
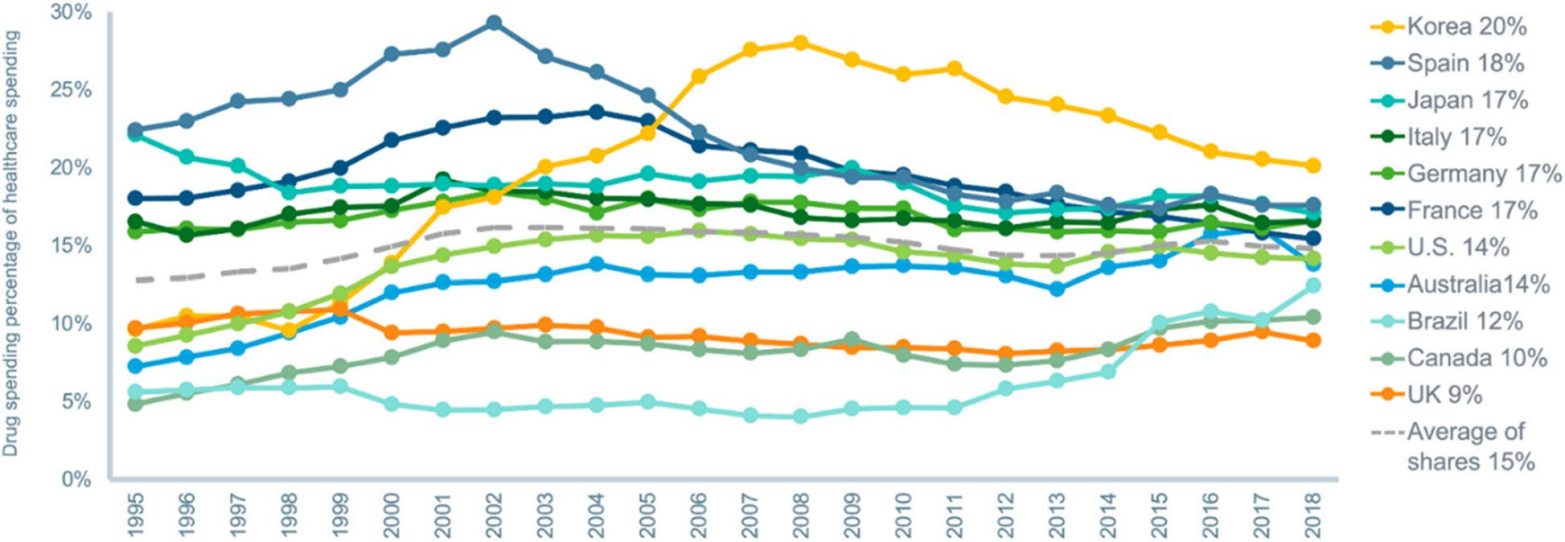
Drug spending percentage of healthcare spending in real purchasing power parity 2020$, 1995–2018. Source: IQVIA Institute for Human Data Science [11]

In a study conducted by IQVIA, on average, drug expenditure was shown to constitute approximately 15% of total healthcare expenditure across eleven major markets (the USA, Japan, Germany, France, Italy, Spain, the UK, Brazil, Canada, South Korea, and Australia), fluctuating within a relatively narrow range of 9–20% [11]. This proportion has remained relatively stable over time (Fig. 6). These eleven markets represent a diverse array of health system structures and financing models. Despite their disparities, it is striking that these countries exhibit relatively analogous patterns and trends in medicine expenditure.

The highest area of healthcare spend in most countries is that spent on hospitals, accounting for 37% in the EU in 2020 and 31% in the USA in 2021 [58, 59]. Despite the fact that less than 15% of healthcare expenditures are allocated to prescription drugs, the majority (nearly 50%) of value analyses (i.e., cost-effectiveness analyses) are performed on medicines [60, 61]. In contrast, surgical or medical procedures were evaluated in only 22% of CEAs [60, 61]. Optimizing health gains for our population requires efficient allocation of resources across both drug and non-drug health technologies. Lack of information on the cost-effectiveness of non-drug interventions can result in less efficient allocation of healthcare resources. This is further compounded by the fact that, as noted above, while spending on biopharmaceutical products will eventually be reduced as patented medicines become generic, the same is not true for hospital spending. The inefficient allocation could arise due to at least two potential reasons: firstly, non-drug procedures may be over-utilized despite their cost-ineffectiveness (e.g., when the prescribing physician stands to personally profit from performing the procedure, thereby potentially biasing their judgment), and, secondly, procedures often lack the outcomes data that the FDA demands of medicines as a condition of approval, so even implicit cost-effective analysis may be impossible, let alone a formal CEA.

Consider that when some medications are prescribed and adhered to, healthcare spending declines by lowering emergency admissions. Eleven such medications reduce emergency admissions by hundreds of thousands annually [62]. These drugs essentially displace a non-genericizable service with a genericizable medicine, a logical way to save money in the long run [63], and yet, if one only scrutinizes the drug’s temporary branded cost without considering the alternative of forever relying on emergency department services to manage patients, society could easily end up misallocating resources away from developing such a medicine. Reasons for not performing value assessments on hospital procedures may include a lack of randomized controlled trial data which are important inputs into cost-effectiveness analyses. However, this and other reasons are not insurmountable, and real-world data can provide important information on the effectiveness of an intervention potentially through time series analyses if comparators are not obvious [64–66]. Comprehending the potential cost-effectiveness of all varieties of healthcare interventions is paramount for enhancing the efficiency of healthcare systems and optimizing the health of populations.

## The use and consequences of limited economic evaluation of new medicines

A somewhat speculative argument can be made that the widespread application of standard CEA has an economic effect and a subtle political effect with potentially large unintended adverse consequences. First, it may be that R&D spending on the development of new medicines is distorted by suppressed incentives due to certain payors not considering the full societal benefits of medicines. Essentially, because some developed countries are using CEA as a bargaining tool to secure lower prices that are actually below what their citizens would be able and willing to pay, they are both free riders on the global R&D investment and possibly causing some innovation to go unfunded [67]. The political effect is that these underestimations of value based on conventional CEA and lower prices outside the USA have been used to argue that Americans are paying more than they should truly be willing to pay. As a result, we would argue, the new US Inflation Reduction Act has been passed as a toe in the price-control water, with an inadequate understanding of what full immersion will imply.

Countries that rely on central planning and traditional CEA as their justification for capping the prices may be using it as an excuse to pay less. What can be harmful about these countries using traditional CEA to justify their unwillingness to pay anywhere close to US prices (or else to limit their patients’ access, thereby cutting the revenues they contribute) is that these analyses may give the US public, policymakers, physicians, and payers the impression that medicines are not worth what the US is paying for them. Traditional CEA can thus give the impression that the US market-based system is broken and in need of a regulatory fix, namely price controls. But when viewed in terms of aggregate impact, a good case can be made [57] that the US’s patent-based, market-based competitive framework is working reasonably well, despite an imperfect national insurance system, to bring about affordable biomedical innovation that benefits not only US citizens, but everyone worldwide. Should US policymakers impose price controls on novel medicines in a misguided effort to spare US payers from overpaying, they would be undermining innovation that would be cost-effective for the US and beneficial globally.

The IRA is a mix of constructive and counterproductive reforms. Reforming insurance to lower out-of-pocket costs for patients makes sense. Subjecting biologics to price controls 13 years after they launch will help ensure that these types of medicines, which do not lend themselves to the same genericization process that renders small molecules inexpensive after patent expiration, abide by the intent of the patent system to see old inventions drop in price. But the aggressive price controlling of small molecule and other drugs approved via the NDA regulatory pathway just 9 years after they launch does not fix any market failure but rather breaks what is already working and therefore merits a rethink.

What then is the role of traditional CEA? Because it seems to be neither comprehensive enough for assessing societal value nor streamlined enough to be a purely budgetary instrument, the utility of traditional CEA is unclear to us. We would argue that the burden of establishing its utility lies with its practitioners. Valuing something is not the same as paying for it. We should appreciate the value of everything precisely so that we do not mistakenly refuse to pay for or invest in something that is well worth having. For example, we must value water even if it is inexpensive so that we do not find ourselves 1 day without it. Similarly, we should value medicines fully even if market-based price competition allows society to enjoy the benefits of many medicines at prices far below their societal value. Traditional CEA omits too much to inform efficient resource allocation and therefore should not remain, in its current form, the accepted HTA tool for making societal resource allocation decisions about innovative medicines, as it is in several leading and influential developed countries.

## Conclusions

Virtually, nothing about healthcare is affordable without private or public insurance. In countries like the USA, prices that some patients pay for treatments are set by their insurance plans. When an insured patient cannot afford a medicine that is medically or clinically appropriate for them, it may be because their insurance has rendered it unaffordable by charging excessive out-of-pocket costs. Affordability for these patients would be best solved by lowering out-of-pocket costs. In that case, society would be spreading the cost of medicines for patients across premiums paid by everyone. This is the case in many European countries where patients have to spend very little out of pocket on treatments their governments have decided merit coverage.

It is reasonable for society to want to be sure that it is getting value for its money. Just by (a) recognizing that most medicines will go generic, (b) can help not only the patient but also caregivers, (c) can restore productivity, and (d) offer system-wide scientific spillovers, we can appreciate that medicines can be worth more than traditional CEA would have us believe. Adding just a few of the commonly overlooked values of medicines to a CEA reveals that many countries have long been negotiating the prices of branded medicines not down to an upper limit of cost-effectiveness but well below their societal value. That only further underscores that the way to make cost-effective medicines affordable to US patients is through insurance reform that lowers out-of-pocket costs.

Central planners—inside and outside the USA—would be wise to subject more than just medicines to generalized, comprehensive CEA. Were they to do that, they would see that the rising cost of healthcare services means that managing the ravages of Alzheimer’s disease in hospitals and nursing homes for an increasing number of aging citizens represents a tsunami of cost and suffering, not just for patients but for caregivers and the taxpaying public. Then, just as many countries have realized the long-term benefits of investing more in clean energy by paying more for those technologies, they might recognize the benefits of investing in incentives for biomedical R&D to avert Alzheimer’s and other diseases, by paying more of what today’s medicines are worth. The larger the perceived market for cost-effective products, the more the private sector will strive to serve that market with more such cost-effective solutions, in the USA and globally.

## Data Availability

Not applicable.

## Abbreviations

BLA: Biologics License Application
CADTH: Canadian Agency for Drugs and Technologies in Health
CEA: Cost-effectiveness analysis
EU: European Union
FDA: Food and Drug Administration
G-BA: Gemeinsamer Bundesausschuss
GDP: Gross domestic product
HAS: Haute Autorité de Santé
HIV: Human immunodeficiency virus
HCV: Hepatitis C virus
HTA: Health technology assessment
ICER: Incremental cost-effectiveness ratio
IRA: Inflation Reduction Act
ISPOR: Professional Society for Health Economics and Outcomes Research
NDA: New drug application
NICE: National Institute of Health and Care Excellence
PBAC: Pharmaceutical Benefits Advisory Committee
PBM: Pharmacy benefit manager
PPP: Purchasing power parity
QALY: Quality-adjusted life year
R&D: Research and development
US: United States
